# Microemulsion Electrokinetic Chromatography in Combination with Chemometric Methods to Evaluate the Holistic Quality Consistency and Predict the Antioxidant Activity of *Ixeris sonchifolia* (Bunge) Hance Injection

**DOI:** 10.1371/journal.pone.0157601

**Published:** 2016-06-23

**Authors:** Lanping Yang, Xiuman Xie, Jing Zhang, Guoxiang Sun

**Affiliations:** School of Pharmacy, Shenyang Pharmaceutical University, Shenyang 110016, China; Indian Institute of Chemical Technology, INDIA

## Abstract

In this paper, microemulsion electrokinetic chromatography (MEEKC) fingerprints combined with quantification were successfully developed to monitor the holistic quality consistency of *Ixeris sonchifolia* (Bge.) Hance Injection (ISHI). ISHI is a Chinese traditional patent medicine used for its anti-inflammatory and hemostatic effects. The effects of five crucial experimental variables on MEEKC were optimized by the central composite design. Under the optimized conditions, the MEEKC fingerprints of 28 ISHIs were developed. Quantitative determination of seven marker compounds was employed simultaneously, then 28 batches of samples from two manufacturers were clearly divided into two clusters by the principal component analysis. In fingerprint assessments, a systematic quantitative fingerprint method was established for the holistic quality consistency evaluation of ISHI from qualitative and quantitative perspectives, by which the qualities of 28 samples were well differentiated. In addition, the fingerprint—efficacy relationship between the fingerprints and the antioxidant activities was established utilizing orthogonal projection to latent structures, which provided important medicinal efficacy information for quality control. The present study offered a powerful and holistic approach to evaluating the quality consistency of herbal medicines and their preparations.

## Introduction

**Fig 1:**
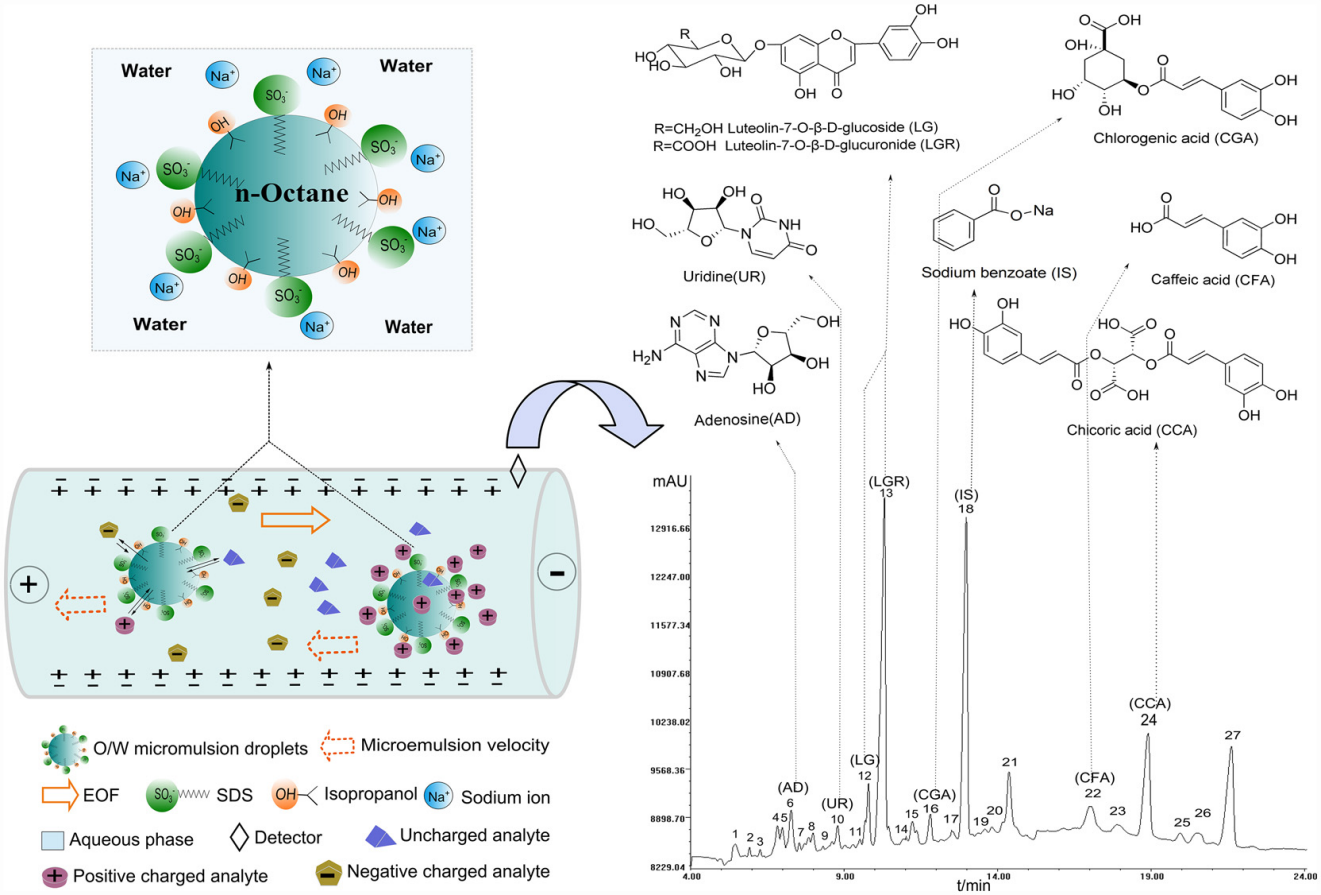
Schematic diagrams of O/W MEEKC separation mechanisms and the electropherogram of ISHI under the optimal MEEKC conditions. i.e., microemulsion solution composed of 0.7% w/v octane, 2.9% w/v SDS, 5.5% w/v 2-propanol, 3.2% w/v methanol, 1.6% w/v acetonitrile and 86.1% v/v 5.7 mM sodium tetraborate-5.7 mM sodium dihydrogen phosphate buffer of pH 9.14.

MEEKC that utilizes microemulsions (MEs) as the background electrophoretic [1], is an electrodriven separation technique based on capillary electrophoresis (CE), and was first introduced by Watarai in 1991 [2]. MEs were commonly made of oil, water, surfactant and cosurfactant by a certain proportion, and could be seen as the expansion of micelles [3]. The unique complex composition of MEs (see Fig 1) makes themselves thermodynamically stable isotropically clear systems. MEs have many characteristic properties, such as thermodynamic stability, optical transparency, and high solubilization capacity [4]. Separation in MEEKC is achieved by the solute mobility and partition coefficients between the aqueous phase and ME droplets, combining electrophoretic and chromatographic behaviors [5]. Compared to capillary zone electrophoresis (CZE) that can only analyze charged substances [6], MEEKC has been proved to be a promising and powerful analytical tool for both charged and neutral or highly hydrophobic and hydrophilic compounds [7] (see Fig 1). MEEKC also has an advantage over micellar electrokinetic chromatography (MEKC) owing to the enhanced solubilization capacity for highly lipophilic compounds and an enlarged migration window [8].

The advantages of MEEKC make it seem to be an appropriate method to analyze complex Traditional Chinese medicine (TCM)/herbal preparations that usually contain a great number of components, which contribute to the therapeutic effects all together with multiple targets [9]. Obviously, it is inadequate to control the quality of the TCM/herbal preparations by quantifying only one or a few marker substances. Fingerprint, especially chromatography fingerprint is a powerful tool for evaluating the quality consistency of complex multi-component herbal preparations [10]. The World Health Organization (WHO) [11], US Food and Drug Administration (FDA) [12] and European Medicine Agency (EMA) [13] have all accepted the chromatography fingerprint method and promoted its use for the quality control of herbal preparations. From 2000, the Chinese State Food and Drug Administration (SFDA) began to require that all injection preparations made from TCM or their raw materials should be standardized by chromatography fingerprint [14]. In recent years, MEEKC method was used for determination and separation analytes in complex natural products, such as flavonoids and phenolic acids [15], polyynes [16], tobacco alkaloids [17], curcuminoids [18] and catechins [19], but few publications have reported on TCM/herbal preparations fingerprint [7]. Furthermore, conventional chromatography fingerprint methods are mostly qualitative based on a simple comparison of similarity of the fingerprints, and often lack the quantitative assessment of the fingerprints [20]. In the present work, a fingerprint of *Ixeris sonchifolia* (Bge.) Hance Injection (ISHI) using MEEKC was first developed (Fig 1), and the holistic quality consistency of ISHI was evaluated by systematic quantitative fingerprint method (SQFM) [21], which can not only qualitatively evaluate the chemical composition, but also provide the quantitative similarity measures for the overall contents of the herbal preparations.

It is well known that the optimization of MEs is the most critical procedure in MEEKC analysis, which has significant influence on the separation. For example, Huang et al. [22] reported that the amounts of cosurfactant, organic modifier and the type of oil were determined as the major impacts on the separation selectivity and resolution of phenolic compounds. However, publications on the optimization of MEs in MEEKC mostly focus on univariate approach [15,23], which ignored the interactions between factors; few reported on multivariate optimization method [24]. Multivariate optimization method, such as the central composite design (CCD), applying a response surface design methodology for complex processes, is a less laborious and time-consuming effective statistical technique than other approaches [25]. However, there are no reported publications on optimization of analytical conditions for MEEKC by CCD method. In the present work, for the first time in the literature, CCD was applied to optimize the MEs in MEEKC.

ISHI, the injection preparation of *Ixeris sonchifolia* (Bge.) Hance (*I*. *sonchifolia*), has been widely used for its anti-inflammatory and haemostatic effects, its influence on improving blood circulation, and potential protection against ischemia brain injury with relatively low side effects [26]. Chemical compositions of *I*. *sonchifolia* are quite complex, including flavonoids, phenolic acids, nucleosides, sesquiterpene lactones, triterpenes and steroids, lignans and amino acids [27]. Published studies have shown that the antioxidant components of *I*. *sonchifolia*, especially flavonoids and phenolic acids, can provide neuro-protective effects against ischemia-induced cellular injury [26,28]. Antioxidants can scavenge free radicals that induce numerous diseases by lipid peroxidation, protein peroxidation, and DNA damage [29], thus, much attention has been paid to the search for the natural antioxidants and the antioxidant property [30]. 2, 2-diphenyl-1-picryldrazyl (DPPH), ABTS^+^· and N,N-dimethyl-p-phenylenediamine dihydrochloride cation radical (DMPD^+^·) are among the most popular spectrophotometric methods for determination of the antioxidant capacity of foods, plant materials and TCM/herbal preparations [29,31]. DPPH is a stable and commercially available radical, providing simple, rapid, sensitive and reproducible procedures, has been the most commonly used method to evaluate the antioxidant activity of flavonoids and phenolic acids in TCM/herbal preparations [32,33]. Considering the main chemical compositions in ISHI are flavonoids and phenolic acids, the antioxidant activity of ISHI was performed by the DPPH assays in this study. Predictive model for the antioxidant activity was also established using the orthogonal projection to latent structures (OPLS) [34] method.

The chief objectives of the present study are to efficiently optimize the MEs in MEEKC with the CCD method, to develop the MEEKC fingerprint and the multiple marker compound analysis under the optimum MEEKC conditions, and to quantitative fingerprint evaluation by SQFM that comprehensively monitor the holistic quality consistency of ISHI. In addition, fingerprint—efficacy relationship between the fingerprints and the antioxidant activities *in vitro* was investigated by OPLS, and the obtained calibration model exhibited good predictive ability.

## Materials and Methods

### Chemicals and reagents

Standards of Adenosine (AD), chlorogenic acid (CGA), caffeic acid (CFA) and sodium benzoate were acquired from the National Institute for the Control of Pharmaceutical and Biological Products (Beijing, China). Chicoric acid (CCA) and luteolin-7-β-D-glucuronide (LGR) were supplied by Chengdu Puri France Science and Technology Development Co., Ltd. (Chengdu, China). Uridine (UR) and 2, 2-diphenyl-1-picryldrazyl (DPPH) were purchased from Sigma Chemical Co. (St. Louis, MO, US). Luteolin-7-glucoside (LG) was provided by Shanghai Winherb Medical Technology Co., Ltd. (Shanghai, China). All the standard compounds have purity above 99%. The structures of seven marker compounds are shown in Fig 1. A total of 28 batches of ISHI (numbered S1-S28, 20ml, apparent concentration = 1.0g/ml), were obtained from different pharmacies in Shenyang, China, which manufactured by Shenyang Shuangding Pharmaceutical Co., Ltd. (Manufacturer A, including S1~S23) and Tonghua Huaxia Pharmaceutical Co., Ltd. (Manufacturer B, including S24~S28), respectively. Methanol (HPLC grade) and acetonitrile (HPLC grade) were purchased from Yuwang Industry Co., Ltd. (Shandong, China). Sodium dodecyl sulfate (SDS) was obtained from Tianjin Bodi Chemical Co., Ltd. (Tianjin, China). Octane and 2-Propanol were acquired from Tianjin Fuyu Fine Chemical Co., Ltd. (Tianjin, China). Sodium tetraborate and sodium dihydrogen phosphate were purchased from Tianjin City Damao Chemical Reagent Factory (Tianjin, China). All other chemicals were reagent-grade.

### Apparatus

All MEEKC experiments were carried out on an Unimicro CE system (HyperQuan, Inc. USA) equipped with a VUV.20 UV detector. Data acquisition were controlled by the Johnson Chromato-Station (Johnson Technology). Separations were performed in an uncoated fused silica capillary (Yongnian Optic Fiber Factory, Hebei, China) with dimensions of 75 μm id ×55 cm (40 cm to detector). The antioxidant activity assay was performed on a 722S spectrophotometer (Shanghai Precision Instrument Co., Ltd., Shanghai, China).

### Electrophoretic conditions

The background electrophoretic buffer was prepared following an optimized method: mixing 0.7% w/v octane, 2.9% w/v SDS, 5.5% w/v 2-propanol, 3.2% w/v methanol, 1.6% w/v acetonitrile and 86.1% v/v 5.7 mM sodium tetraborate-5.7 mM sodium dihydrogen phosphate buffer of pH 9.14, then sonicated the mixture for 30 min until homogeneous, filtered the MEs through a 0.22μm polyvinylidene fluoride (PVDF) filter before use. Prior to its first use, the capillary was sequentially conditioned by rinsing with 1.0 M sodium hydroxide for 5 min, deionized water for 5 min, 0.1 M sodium hydroxide for 10 min, deionized water for 5 min and running buffer for 10 min. Between two runs, the capillary was flushed with deionized water for 3 min and running buffer for 3 min. After the last run of each day, capillaries were washed with 0.1 M sodium hydroxide for 10 min and deionized water for10 min. The sample solutions were introduced into the capillary by hydrodynamic injection (10 cm height) for 20 s (amount to 28 nL). The separation of ISHI was conducted at 16 kV at 25°C, and the detection wavelength was set at 210 nm.

### Preparation of standard and sample solutions

Standards, UR, AD, CGA, CFA, CCA, LGR and LG were accurately weighed and dissolved in methanol/water (3:2, v/v) as mixed standard stock solution. Sodium benzoate standard was accurately weighed and dissolved in methanol/water (3:2, v/v) as internal standard (IS) solution. Six concentration levels of mixed standard solutions were prepared by dilution with methanol/water (3:2, v/v). Then, 9 mL of the mixed standard solutions and 1 mL of the IS solution were mixed in a 10 mL volumetric flask for the calibration curves, and stored at 4°C prior to use.

Each tested ISHI sample was filtered through 0.45 μm Millipore filters (Beijing Sunrise T&D Company, China) prior to use.

#### Methodology validation

In MEEKC analysis, injection errors and migration fluctuation could be effectively reduced or even eliminated by adding IS in the test samples. Taking sodium benzoate IS as a reference, the relative migration times and the relative peak areas of the co-possessing peaks were calculated to estimate the repeatability, stability, and precision tests. The repeatability was determined by analyzing six individual sample solutions. The stability was evaluated by analyzing a single sample solution stored at room temperature for 0, 2, 4, 8, 16, and 24 h. Intra-day and inter-day precision of the method were validated by nine replicate injections of the standard mixture solution three times a day over three consecutive days.

### Antioxidant activity assay

Total antioxidant activity of ISHI was determined by the DPPH radical scavenging assay according to Bhandari et al. [35] with slight modification. Briefly, 2 mL of ISHI sample solution was added to 2 mL of the 0.127 mM DPPH solution (in methanol), and then diluted in methanol to various concentrations. The absorbance was measured at 517 nm against a blank after those solutions stand in the dark for 40 minutes. All tests were performed in triplicates. The radical scavenging capacity is expressed as percent inhibition and calculated using the following equation: %inhibition = [(*A*_*control*_ − *A*_*sample*_/*A*_*control*_)] × 100, where *A*_*control*_ is the absorbance of the negative control and *A*_*sample*_ is the absorbance at the presence of the ISHI sample. The total antioxidant activity was calculated by plotting the percent inhibition against the sample concentration, and represented as IC_50_ values, i.e. the concentration of samples required to scavenge 50% of DPPH radicals, the lower IC_50_ value, the stronger antioxidant activity.

### Data analysis

All the original data acquired were processed by Johnson Chromato-Station (Johnson Technologies). An in-house developed software “Digitized Evaluation System for Super-Information Characteristics of TCM Fingerprints 4.0” (software certificate No. 0407573, China) was used to evaluate the holistic quality consistency. The Design-expert software (Version 8.05, Stat-Ease, Inc., United States) and the SIMCA-P+ software (Version 13.0, Umetrics, Umea, Sweden) were used for central composite design and chemometric analysis, respectively.

#### OPLS analysis

OPLS, a generic preprocessing method for multivariate data, can reduce the complexity of models and preserve the ability of prediction at the same time [34]. In this study, OPLS model was constructed to characterize the correlation between total antioxidant activity and chemical content of the ISHI samples. Areas of 27 characteristic peaks as the descriptor matrix *X* and 1/IC_50_ values as the response matrix *Y*. The confidence level was set at 95% with the SIMCA-P+ software (Version 13.0, Umetrics, Umea, Sweden).

### Theory

The sample fingerprint (SFP) and reference fingerprint (RFP) vectors are defined as $\vec{\text{x}}=\left(x_{1},x_{2,}\cdots ,x_{n}\right)$ and $\vec{\text{y}}=\left(y_{1},y_{2,}\cdots ,y_{n}\right)$, where *x*_*i*_ and *y*_*i*_ are the peak areas of all the co-possessing peaks in the SFP and RFP vectors, respectively. Calculating the cosine of the angle between SFP and RFP vectors provides qualitative similarity (*S*_*F*_) as defined in Eq (1). In order to limit the influence of the large peaks and ensure an equal weight for each peak, the SFP ($\vec{x}$) and RFP ($\vec{y}$) vectors are transformed to ${\vec{P}}_{s}=\left(\frac{x_{1}}{y_{1}},\frac{x_{2}}{y_{2}},\cdots \frac{x_{n}}{y_{n}}\right)$ and ${\vec{P}}_{0}=\left(1,1,1\cdots 1\right)$, respectively. The cosine of the angle between the vectors ${\vec{P}}_{o}$ and ${\vec{P}}_{s}$ is defined as the qualitative ratio similarity ($S_{F}^{'}$), as calculated by Eq (2). Macro qualitative similarity (*S*_*m*_), a qualitative parameter be obtained by averaging *S*_*F*_ and $S_{F}^{'}$ as showed in Eq (3), can accurately describe the resemblance in terms of the distribution and number of fingerprints between SFP and RFP. For quantitative assessment of the fingerprints, the projection of $\vec{x}$ to $\vec{y}$ is defined as projection content similarity (*C*) as calculated in Eq (4). The quantitative similarity (*P*) is the ratio of the total content corrected by the qualitative similarity factor *S*_*F*_, as showed in Eq (5). Combining the above two quantitative properties yields macro quantitative similarity (*P*_*m*_) as defined in Eq (6), which is a measure to monitor the overall content of chemical components in the sample fingerprint. Finally, a fingerprint leveling coefficient (*α*), as defined in Eq (7), is another quantitative parameter that can detect the difference between SFP and RFP.

$$S_{F}=\text{cos}\theta =\frac{\sum_{i=1}^{n}x_{i}y_{i}}{\sqrt{\sum_{i=1}^{n}x_{i}^{2}}\sqrt{\sum_{i=1}^{n}y_{i}^{2}}}$$ (1)

$$S_{F}^{'}=\text{cos}\theta^{'}=\frac{\sum_{i=1}^{n}\frac{x_{i}}{y_{i}}}{\sqrt{n\sum_{i=1}^{n}{\left(\frac{x_{i}}{y_{i}}\right)}^{2}}}$$ (2)

$$S_{m}=\frac{1}{2}\left(S_{F}+S_{F}^{'}\right)=\frac{1}{2}\left(\frac{\sum_{i=1}^{n}x_{i}y_{i}}{\sqrt{\sum_{i=1}^{n}x_{i}^{2}}\sqrt{\sum_{i=1}^{n}y_{i}^{2}}}+\frac{\sum_{i=1}^{n}\frac{x_{i}}{y_{i}}}{\sqrt{n\sum_{i=1}^{n}{\left(\frac{x_{i}}{y_{i}}\right)}^{2}}}\right)$$ (3)

$$C=\frac{\sum_{i=1}^{n}x_{i}y_{i}}{\sum_{i=1}^{n}y_{i}^{2}}\times 100\%$$ (4)

$$P=\frac{\sum_{i=1}^{n}x_{i}}{\sum_{i=1}^{n}y_{i}}S_{F}\times 100\%$$ (5)

$$P_{m}=\frac{1}{2}\left(C+P\right)=\frac{1}{2}\left(\frac{\sum_{i=1}^{n}x_{i}y_{i}}{\sum_{i=1}^{n}y_{i}^{2}}+\frac{\sum_{i=1}^{n}x_{i}}{\sum_{i=1}^{n}y_{i}}S_{F}\right)\times 100\%$$ (6)

$$\alpha =\left|1-\frac{P}{C}\right|$$ (7)

Accordingly, the quality evaluation method in terms of *S*_*m*_, *P*_*m*_ and α is named as SQFM [21], by which TCM and herbal preparations quality can be classified into 8 grades. The evaluation criteria by SQFM are listed in Table 1. Based on the criteria, all *Sm*, *Pm* and *α* are used together in the rules for classification, and the final quality grade is on the basis of the worst one. For example, if *S*_*m*_ 0.98 (grade 1), *P*_*m*_(%) 101 (grade 1) and *α* 0.04(grade 1), then quality is grade 1; if *S*_*m*_ 0.91 (grade 2), *P*_*m*_(%) 85.5 (grade 3) and *α* 0.06 (grade 2), then quality is grade 3; if *S*_*m*_ 0.88 (grade 3), *P*_*m*_(%) 103 (grade 1) and *α* 0.22 (grade 5), then quality is grade 5. In the evaluation system, grade 1 belongs to the highest quality and grade 8 to the lowest one, and the grades in the range of 1–5 are recognized as qualified.

**Table 1:** The quality grade criteria based on SQFM.

Grade	1	2	3	4	5	6	7	8
*S*_*m*_≥	0.95	0.90	0.85	0.80	0.70	0.60	0.50	<0.50
*P*_*m*_(%)∈	95∼105	90∼110	80∼120	75∼125	70∼130	60∼140	50∼150	0∼∞
*α*≤	0.05	0.10	0.15	0.20	0.30	0.40	0.50	>0.50
Quality	Best	Better	Good	Fine	Moderate	Common	Defective	Inferior

## Results and Discussion

### Optimization of MEEKC condition

#### Preliminary investigations

**Fig 2:**
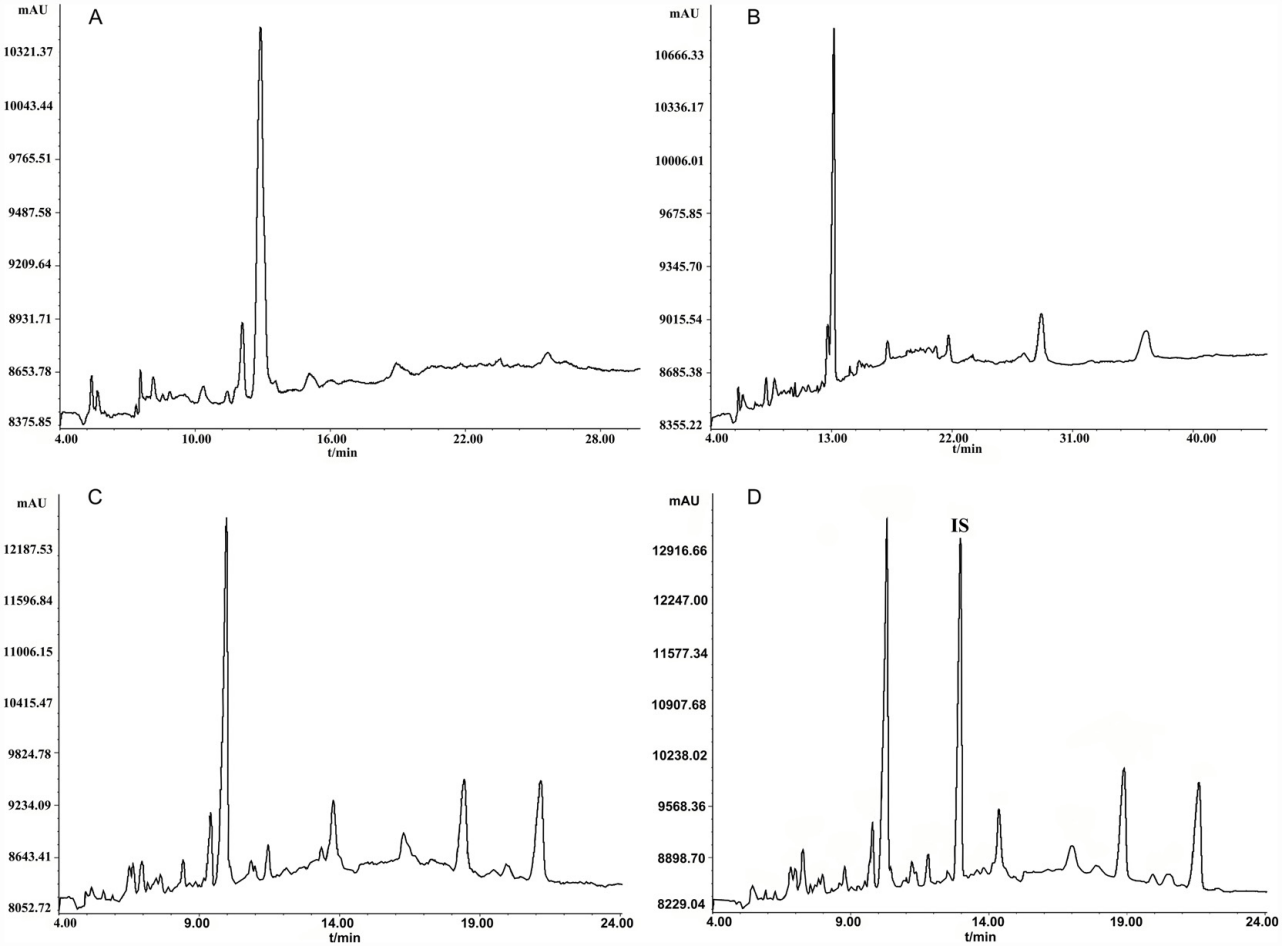
Electropherograms. CZE (A), MEKC (B), typical MEEKC chromatograms of sample solution without (C) and with (D) sodium benzoate as an internal standard (IS) under the optimized MEEKC conditions.

Preliminary experiments with different buffers showed that CZE (capillary zone electrophoresis, as described in Method A in S1 File) (Fig 2A) and MEKC (micellar electrokinetic chromatography, as described in Method B in S1 File) (Fig 2B) gave poor resolution and long migration time, while MEEKC provided a relatively better separation in shorter migration time. Thus, optimization conditions for MEEKC were investigated subsequently. The mixture of 20 mM sodium tetraborate and 20 mM sodium dihydrogen phosphate was chosen as the aqueous buffer because it provided a relatively better separation. Soon afterwards, the pH value (7 to 11) of the buffer was investigated and pH 9.14 seen as the optimum. The effects of voltage (10 kV~20 kV), injection time (10 s~30 s) and range (0.01~1.00) were studied, where the optimum applied voltage, injection time and range condition were set at 16 kV, 20 s and 0.05, respectively. In this study, octane, n-hexane, ethyl acetate, cyclohexane and octanol were examined as the oil phase in the MEs droplet, where octane was chosen as the oil phase for the better repeatability and the more stable baseline. SDS was selected as the surfactant. The cosurfactants of butanol and 2-propanol were studied and 2-propanol was employed as cosurfactant for a better separation resolution. Methanol and acetonitrile were selected as organic modifiers to improve the separation of analytes.

#### Central composite design (CCD)

**Fig 3:**
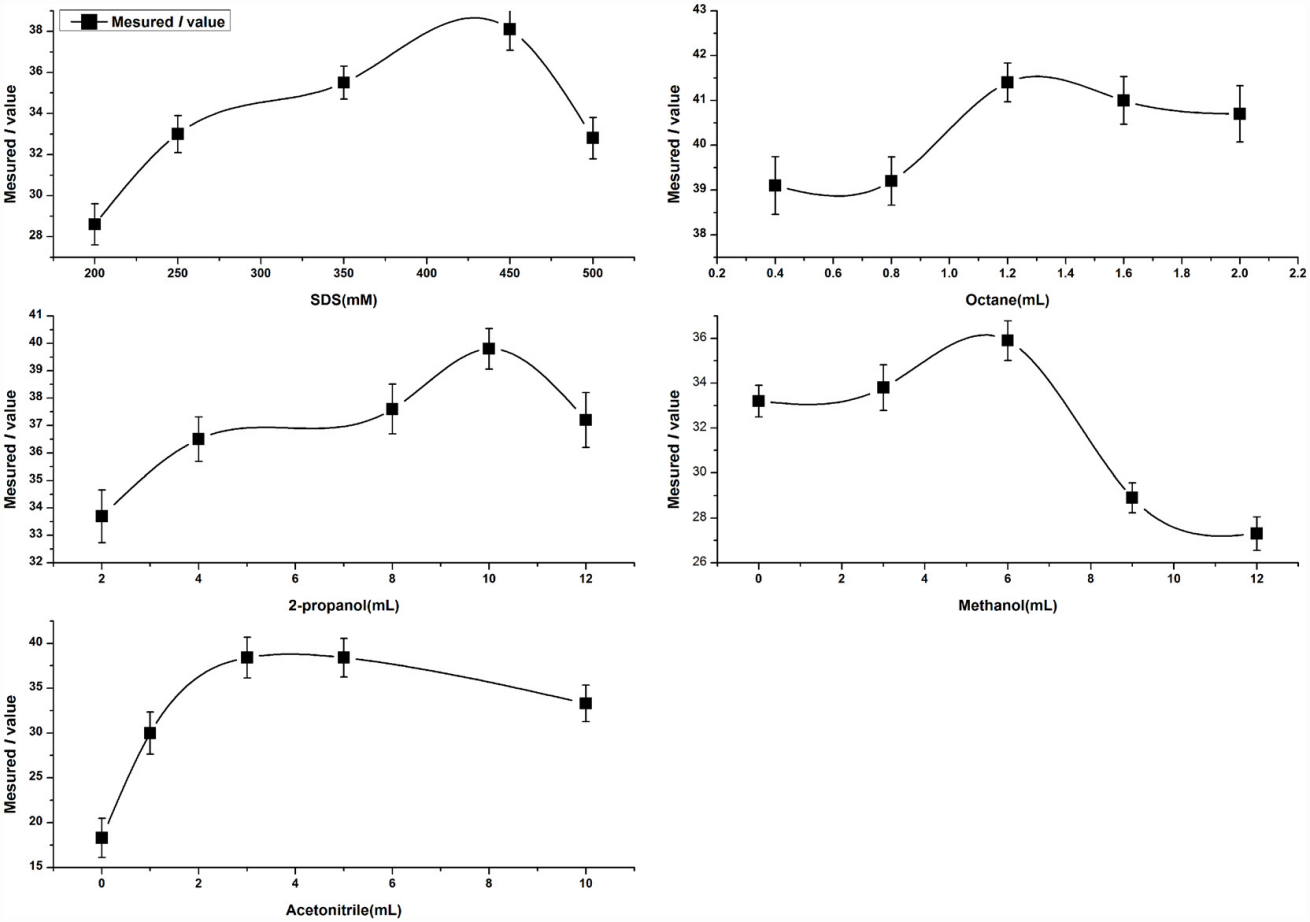
Effects of five crucial investigated variables on the measured *I* value. The concentrations were varied in the range of 200–500 mM SDS (A), 0.4–2.0 mL octane (B), 2.0–12.0 mL 2-propanol (C), 0.0–12.0 mL methanol (D) and 2.0–12.0 mL acetonitrile (E).

CCD was performed to optimize the MEs conditions of five crucial experimental parameters, i.e. the concentration of SDS, octane, 2-propanol, methanol and acetonitrile that displayed much more pronounced effects on the performance of separation compared to the voltage and injection condition. The resolution index (*I*) [36] was adopted as the separation efficiency response of the CCD. $I=-\sum_{i=1}^{n}P_{i}lnP_{i}lnA_{i}$, where *A*_*i*_ is the peak area, *P*_*i*_ is the normalized value of the peak area. *I* represents the resolution, effective signal amount and degree of fingerprint signal uniformity. The higher *I* value, the better experimental condition. From the effects of the five crucial investigated variables on the measured *I* value in Fig 3, the following upper and lower limits were selected: the concentration of SDS (*X*_*1*_) range from 250 mM to 450 mM, octane (*X*_*2*_) from 0.8 mL to 1.2 mL, 2-propanol (*X*_*3*_) from 4.0 mL to 10.0 mL, methanol (*X*_*4*_) from 1mL to 6.0 mL, and acetonitrile (*X*_*5*_) from 1.0 mL to 3.0 mL. The experimental conditions for CCD and the response results were presented in Table 2. According to the formula [37] of total experimental number, *N* = 2^*k*^ × 1/2 + 2*k* + *c*_*p*_, where *k* is the factor number and *c*_*p*_ is the replicate number of the central point, total 36 experiments (*k = 5*) were involved in the matrix of CCD including 10 central point (*c*_*p*_ = *10*) to estimate the overall error. The experiments were performed in random order to avoid the systematic errors.

**Table 2:** Experimental parameters for central composite design and the corresponding response results.

No.	X1: SDS (mM)	X2: Octane (mL)	X3: 2-Propanol (mL)	X4: Methanol (mL)	X5: Acetonitrile (mL)	Measured *I* value
1	250.0	0.8	4.0	1.0	3.0	35.1
2	450.0	0.8	4.0	1.0	1.0	37.1
3	250.0	1.2	4.0	1.0	1.0	35.2
4	450.0	1.2	4.0	1.0	3.0	35.8
5	250.0	0.8	10.0	1.0	1.0	35.0
6	450.0	0.8	10.0	1.0	3.0	32.6
7	250.0	1.2	10.0	1.0	3.0	37.7
8	450.0	1.2	10.0	1.0	1.0	36.5
9	250.0	0.8	4.0	6.0	1.0	35.4
10	450.0	0.8	4.0	6.0	3.0	38.4
11	250.0	1.2	4.0	6.0	3.0	35.7
12	450.0	1.2	4.0	6.0	1.0	38.6
13	250.0	0.8	10.0	6.0	3.0	37.6
14	450.0	0.8	10.0	6.0	1.0	35.3
15	250.0	1.2	10.0	6.0	1.0	34.1
16	450.0	1.2	10.0	6.0	3.0	36.7
17	150.0	1.0	7.0	3.5	2.0	35.3
18	550.0	1.0	7.0	3.5	2.0	37.2
19	350.0	0.6	7.0	3.5	2.0	35.3
20	350.0	1.4	7.0	3.5	2.0	35.4
21	350.0	1.0	1.0	3.5	2.0	37.1
22	350.0	1.0	13.0	3.5	2.0	35.6
23	350.0	1.0	7.0	-1.5	2.0	36.6
24	350.0	1.0	7.0	8.5	2.0	38.1
25	350.0	1.0	7.0	3.5	0.0	42.3
26	350.0	1.0	7.0	3.5	4.0	43.9
27–36	350.0	1.0	7.0	3.5	2.0	43.7

The CCD design permits the response surface to be modeled by fitting the second-order regression models, which can be shown as

$$\begin{array}{l} I\;=-47.58+0.14X_{1}+99.14X_{2}+2.92X_{3}+1.97X_{4}+1.69X_{5}+0.01X_{1}X_{2}-2.46*10^{-3}X_{1}X_{3}+1.80*10^{-3}X_{1}\\ X_{4}-6.50*10^{-3}X_{1}X_{5}+0.54X_{2}X_{3}-0.87X_{2}X_{4}-0.02X_{3}X_{4}+0.10X_{3}X_{5}+0.19X_{4}X_{5}-1.86*\\ 10^{-4}X_{1}^{2}-52.03X_{2}^{2}-0.20X_{3}^{2}-0.25X_{4}^{2}-0.14X_{5}^{2}\;\left(R^{2}=0.9933,\;P<0.0001\right) \end{array}$$ (8)

Where *I* is the measured response, *X*_1_ to *X*_5_ represents the SDS, octane, 2-propanol, methanol and acetonitrile, respectively. The determination coefficient (*R*^2^ = 0.9969) was shown by ANOVA of the quadratic regression model, indicating that only 0.31% of the total variations were not explained by the model. The value of the adjusted determination coefficient (*R*^2^ = 0.9933) also confirmed that the model was highly significant. At the same time, a very low value 0.79% of coefficient of variation (CV) clearly indicated a very high degree of precision and a good deal of reliability of the experimental values. The model was found to be adequate for prediction within the range of experimental variables. The regression coefficient values of Eq (8) were listed in Table 3, indicating that the standard errors of all the coefficient estimates were less then 0.1, and all of the coefficients were significant, with very small *P*-value (*P* < 0.05). The effects of every two factors on *I* were intuitively presented by 3D response surface plots (depicted in Figure A in S1 File). Five variables at the optimum value of average *I* (43.8) proposed were determined. And the following optimum MEEKC conditions have been selected: the concentration of SDS, 346 mM; octane, 1.0 mL; 2-propanol, 7.0 mL; methanol, 4.0 mL; and acetonitrile, 3.0 mL.

**Table 3:** Regression coefficient of the predicted quadratic polynomial model.

Factor	Coefficient Estimate	Standard Error	*t* ratio	*P*-value Prob. > F
Intercept	-47.58	0.095	1.01	< 0.0001
*x*_1_	0.14	0.062	2.13	< 0.0001
*x*_2_	99.14	0.062	8.57	0.0163
*x*_3_	2.92	0.062	0.46	< 0.0001
*x*_4_	1.97	0.062	1.93	< 0.0001
*x*_5_	1.69	0.062	3.70	0.0017
*x*_1_ * *x*_2_	0.01	0.076	3.46	0.0016
*x*_1_ * *x*_3_	-2.46*10^−3^	0.076	0.64	< 0.0001
*x*_1_ * *x*_4_	1.80*10^−3^	0.076	2.10	< 0.0001
*x*_1_ * *x*_5_	-6.50*10^−3^	0.076	0.60	< 0.0001
*x*_2_ * *x*_3_	0.54	0.076	3.06	0.0006
*x*_2_ * *x*_4_	-0.87	0.076	0.47	< 0.0001
*x*_3_ * *x*_4_	-0.02	0.076	0.07	0.0254
*x*_3_ * *x*_5_	0.10	0.076	3.13	0.0008
*x*_4_ * *x*_5_	0.19	0.076	2.06	< 0.0001
*x*_1_^2^	-1.86*10^−4^	0.054	0.88	< 0.0001
*x*_2_^2^	-52.03	0.054	0.90	< 0.0001
*x*_3_^2^	-0.20	0.054	0.88	< 0.0001
*x*_4_^2^	-0.25	0.054	0.86	< 0.0001
*x*_5_^2^	-0.14	0.054	0.12	0.0167

#### Model validation

To evaluate the model performance, measured and predicted *I* values in optimum BGE were compared. The standard error between measured *I* value (43.87±0.07, n = 3) and predicted *I* value (43.79) was 0.18%±0.16 (n = 3), demonstrating that the used optimization strategy was therefore validated. Based on the above results, the optimized MEEKC conditions composed of 0.7% w/v octane, 2.9% w/v SDS, 5.5% w/v 2-Propanol, 3.2% w/v methanol, 1.6% w/v acetonitrile, 86.1% v/v 5.7 mM sodium tetraborate-5.7 mM sodium dihydrogen phosphate buffer of pH 9.14.

Under the optimized MEEKC conditions, the optimal electropherograms were observed, which possessed efficient separation characteristics as well as abundant fingerprint information (Fig 2C). Sodium benzoate was chosen as the internal standard (IS) with good separation characteristics and appropriate migration time (Fig 2D).

### Quantitative analysis of seven marker compounds

In typical MEEKC fingerprints (Fig 1), seven peaks 6, 10, 12, 13, 16, 22 and 24 were identified as adenosine (AD), uridine (UR), luteolin-7-glucoside (LG), luteolin-7-β-D-glucuronide (LGR), chlorogenic acid (CGA), caffeic acid (CFA) and chicoric acid (CCA), respectively, using standard addition method. Peak 18 was the IS sodium benzoate.

#### Methodology validation of quantitative analysis

Under the optimized MEEKC conditions, the calibration curves were constructed by plotting relative peak area (sodium benzoate as internal standard) vs. the concentration from single measurements (n = 1) and no outliers were removed. The constructed calibration curves presenting good linearity (*R*^2^≥0.9992) for all the marker compounds in the targeted concentration ranges (Table 4), in agreement with the reported literature (*R*^2^≥0.9996) [38]. The limit of detection (LOD, S /N = 3) and the limit of quantification (LOQ, S /N = 10) were determined in the range of 0.20–0.39 μg·mL^-1^ and 0.69–1.23 μg·mL^-1^ for the marker compounds. The RSDs of the relative migration time and the relative peak area of the seven marker standards were, respectively, less than 0.8% and 4.5% for the stability, repeatability, and precision tests. The spiked recovery was used to assess method accuracy and their recoveries were between 98.6±2.3 to 101.6±3.1 (%, n = 6). These results demonstrated that the developed method was precise, accurate and sensitive enough for the quantitative analysis requirements.

**Table 4:** Results for calibration curve, R^2^ and linear range of seven marker compounds.

Compound^a)^	Calibration curve^b)^	R^2^	LOD (μg·mL^-1^)	LOQ (μg·mL^-1^)	Linear range (μg·mL^-1^)
AD	*y* = 0.0398*x* - 0.0492	0.9992	0.39	1.23	1.45–8.72
UR	*y* = 0.0279*x* + 0.012	0.9994	0.23	0.98	1.56–15.56
LG	*y* = 0.0198*x* + 0.0244	0.9996	0.28	0.99	3.22–16.11
LGR	*y* = 0.006*x* + 0.3138	0.9993	0.31	1.08	31.9–159.5
CGA	*y* = 0.0223*x* + 0.0052	0.9995	0.24	1.01	3.16–18.95
CFA	*y* = 0.0335*x* - 0.1597	0.9994	0.20	0.69	8.00–40.00
CCA	*y* = 0.0039*x* + 0.3048	0.9997	0.22	0.96	5.16–154.8

^a)^ UR, Uridine; AD, Adenosine; CGA, Chlorogenic acid; CFA, Caffeic acid; CCA, Cichoric acid; LGR, Luteolin-7-β-D-glucuronide; LG, Luteolin-7-glucoside.

^b)^ *y* is the peak area, *x* is the concentration injected (μg·mL^-1^)

#### Sample analysis

The content of the seven marker compounds was simultaneously determined in 28 samples using the established calibration curves (Table 4). From the results presented in Table 5, LGR with the contents varying from 68.99 to 152.33 mg·L^-1^ in 28 samples, with almost 2.2-fold variation, was found to be the main component in ISHI, which was consistent with previous literature by HPLC method [38,39]. Similar variation could also be found for the other components. The reasons for the variation of the contents can be the difference of raw herbs variability caused by a wide range of factors or variability in manufacturing processes.

**Table 5:** Overview of the contents of seven marker compounds and the IC_50_ values for 28 batches of ISHIs.

Sample	Content (mg·L^-1^)								IC_50_ (mg·mL^-1^)
	AD	UR	LG	LGR	CGA	CFA	CCA	*P*_7C_^a)^	
S1	1.61	3.07	7.12	93.29	4.29	15.26	46.16	24.40	2.85
S2	1.98	3.47	5.84	68.99	6.04	14.01	26.01	18.05	3.21
S3	5.82	4.03	4.37	97.13	5.10	12.12	20.63	21.32	3.21
S4	2.89	4.30	5.68	90.30	4.95	17.94	31.89	22.57	2.75
S5	3.55	2.24	14.27	99.30	9.08	27.28	42.08	28.26	2.82
S6	4.85	2.55	5.65	100.20	5.10	12.14	27.52	22.57	3.17
S7	3.78	3.52	8.52	72.69	10.43	19.25	46.55	23.53	2.66
S8	6.83	4.24	9.37	79.59	7.63	12.61	24.81	20.73	3.06
S9	6.30	2.60	8.33	94.06	6.04	18.18	44.40	25.70	3.16
S10	1.49	2.93	7.10	95.36	5.09	13.59	38.01	23.37	3.04
S11	6.90	4.57	11.02	111.57	9.91	19.63	43.97	29.65	2.99
S12	4.56	2.17	5.19	103.65	5.10	11.84	40.15	24.67	3.02
S13	2.88	2.47	5.77	99.58	9.31	16.37	33.86	24.32	2.71
S14	3.41	3.06	7.81	130.12	8.74	17.46	31.93	28.93	2.83
S15	6.26	3.26	5.93	85.62	5.65	11.85	18.93	19.64	3.38
S16	3.85	2.50	3.52	95.84	4.61	11.43	28.77	21.50	3.11
S17	5.92	3.99	10.43	113.42	9.00	11.92	37.96	27.52	3.14
S18	4.65	3.50	6.05	101.12	5.00	11.80	27.48	22.80	3.09
S19	4.17	3.37	7.50	113.03	12.74	19.27	41.53	28.80	2.71
S20	4.01	3.28	6.30	74.64	8.05	12.37	12.82	17.35	3.25
S21	3.46	5.38	7.62	87.69	13.69	13.73	23.18	22.11	3.42
S22	5.97	3.46	14.71	145.71	12.17	28.18	68.26	39.78	2.48
S23	1.43	5.37	12.47	152.34	18.26	21.73	57.41	38.43	3.50
S24	0.43	5.20	8.23	97.33	4.56	14.25	42.27	24.61	2.65
S25	1.73	5.67	6.15	101.44	3.23	11.13	19.79	21.31	3.29
S26	2.58	3.97	5.62	92.26	7.61	17.29	35.90	23.60	2.68
S27	1.85	6.02	8.85	79.65	5.29	21.72	42.74	23.73	2.36
S28	0.44	5.10	9.05	108.63	4.97	9.66	21.29	22.73	3.06

^a)^ the average content of the seven marker compounds

**Fig 4:**
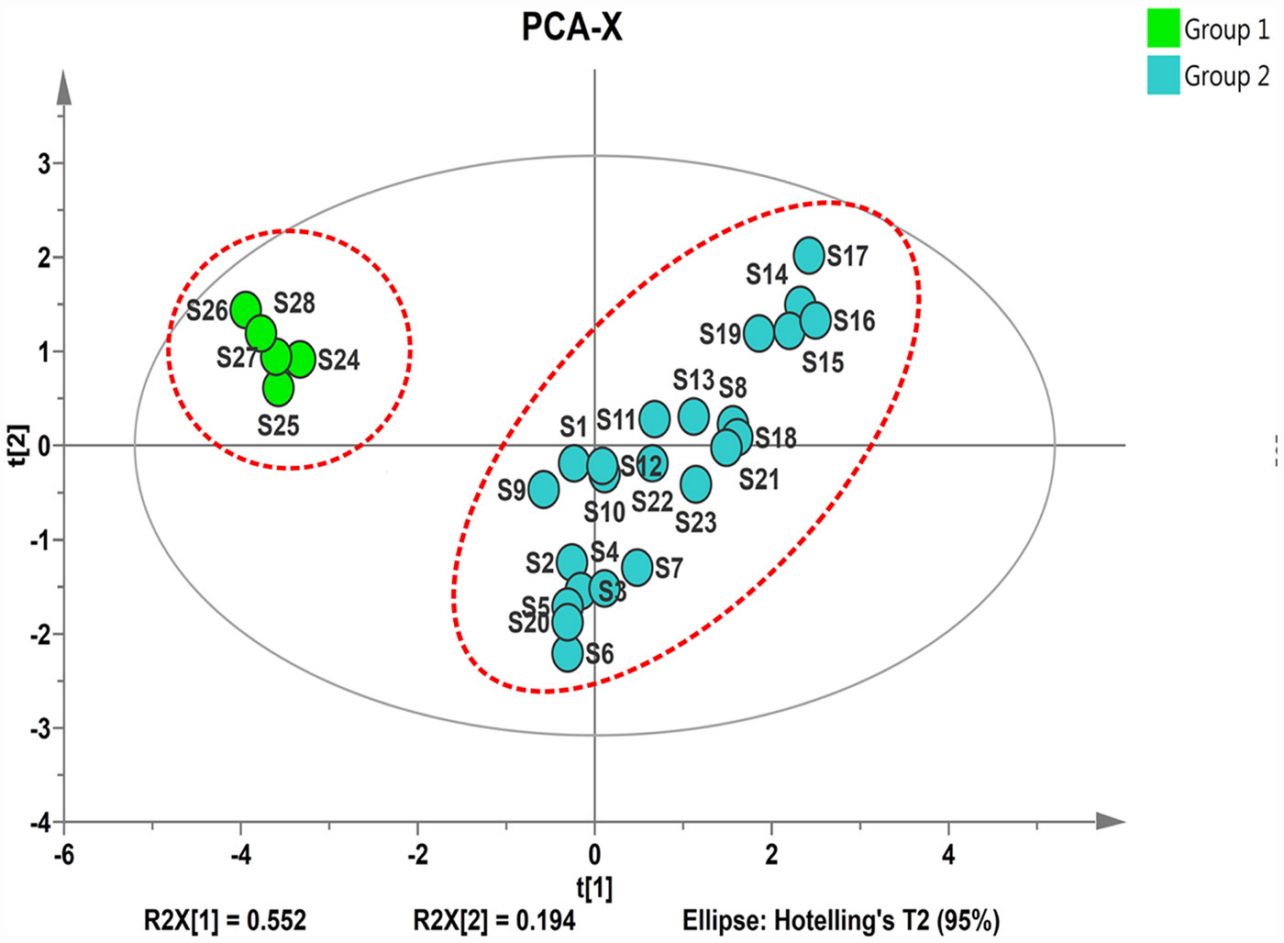
The PCA score plot(A) and Loading pot (B) of seven marker compounds in 28 ISHI samples. Plots given by the SIMCA-P+ software (Version 13.0). The ellipse shows 95% confidence intervals. The abbreviations were the same as in Fig 1.

Principal component analysis (PCA) [10] was carried out using the content of the seven marker compounds in 28 samples to evaluate the discriminating ability of the the marker components. A three-component PCA model was performed with a total variance of 87.0% explained (PC1 = 55.2%, PC2 = 19.4%, and PC3 = 12.5%). In the PCA score plot (Fig 4), 28 samples were clearly divided into two clusters marked as group 1 and 2, respectively. Where group 1 (S24~S28) that all originated from manufacturer A was obviously different from group 2 (S21~S23) that from manufacturer B. Therefore, from the PCA results, we could conclude that the products from the same manufacturer, qualitatively, had relatively good consistency.

### Fingerprint analysis

#### Methodology validation of fingerprint analysis

The optimized MEEKC method was validated in terms of the repeatability, precision, and stability tests. Peak 18 was chosen as the reference peak for its suitable separation with the adjacent peaks and appropriate migration time. RSDs of the relative migration time and the relative peak area of all the analytes, respectively, less than 1.2% and 5.1% for the stability, repeatability, and precision tests, indicating that the experiment was precise and repeatable and the sample was stable during the analytical procedure.

#### Evaluation of fingerprints by SQFM

The developed fingerprint method was successfully applied to 28 samples, and the typical MEEKC fingerprints at 210 nm are shown in Fig 1, which contained 27 co-possessing fingerprints in 24 minutes, better than the reported literature with 24 co-possessing fingerprints in 35 minutes by the CZE method [40]. The quality evaluation results of 28 samples and the reference fingerprint (constructed by taking the average of all the sample chromatograms) based on SQFM (see Table 6) were obtained by importing the sample fingerprint signals to the in-house software mentioned above.

**Table 6:** Evaluation results of fingerprints by SQFM.

Para.	S1	S2	S3	S4	S5	S6	S7	S8	S9	S10	S11	S12	S13	S14	S15
*S*_*m*_	0.94	0.95	0.95	0.92	0.91	0.95	0.92	0.94	0.93	0.93	0.94	0.95	0.92	0.95	0.94
*P*_*m*_(%)	95.7	85.5	94.9	94.6	111.3	89.8	95.7	92.5	103.0	91.0	112.3	92.9	98.2	105.0	89.7
*α*	0.09	0.04	0.02	0.03	0.13	0.12	0.01	0.01	0.03	0.11	0.12	0.11	0.05	0.02	0.03
Grade	2	3	2	2	3	3	2	2	2	3	3	3	2	1	3
Quality	better	good	better	better	good	good	better	better	better	good	good	good	better	best	good
Para.	S16	S17	S18	S19	S20	S21	S22	S23	S24	S25	S26	S27	S28	RFP	
*S*_*m*_	0.95	0.98	0.92	0.93	0.96	0.84	0.91	0.91	0.90	0.94	0.88	0.88	0.92	1.00	
*P*_*m*_(%)	86.9	108.1	95.9	106.3	85.3	95.0	124.1	121.7	102.4	89.4	101.8	98.3	87.8	100.0	
*α*	0.14	0.04	0.03	0.02	0.03	0.03	0.00	0.20	0.11	0.13	0.05	0.04	0.13	0.00	
Grade	3	2	2	2	3	4	4	5	3	3	3	3	3	1	
Quality	good	better	better	better	good	fine	fine	moder	good	good	good	good	good	best	

Table 6 shows that except S21, the remaining 27 samples had the *S*_*m*_ values above 0.85 and all of the 28 samples had the *α* values below 0.15, indicating that they were all similar to RFP in the number and distribution of the fingerprints. Based on qualitative parameters *S*_*m*_ and *α*, the quality grades of the 27 samples should be in the range of grade 1~3. However, only 25 samples met the grade, the other two samples, namely S22 and S23 had the grade 4 or 5 in combination with a quantitative similarity parameter *P*_*m*_. This result indicated that although qualitative evaluation (*S*_*m*_ and *α*) was important, quantitative assessment (*P*_*m*_) should not be ignored, because *P*_*m*_ as a quantitative measure is more discriminating and makes a greater contribution to the quality grade. Generally, samples with the grade≤5 were recommended as the qualified ones. Accordingly, in this study, the qualities of 28 samples were all judged as qualified; the holistic quality consistency of the samples from manufacturer B (S24~S28) was better than manufacturer A (S1~S23). From the SQFM results, it was possible to deduce that the products from the same manufacturer, quantitatively, had relatively good consistency. SQFM offers qualitatively and quantitatively assess TCM/HM fingerprints simultaneously, and provides a reliable and feasible means to control the holistic quality consistency of the ISHI preparations.

The relationship between the fingerprint and the quantitative content of the marker compounds was further investigated. A reasonable linear correlation (*R*^2^ = 0.8817) was obtained using the macro quantitative similarity factors *P*_*m*_ calculated with 27 common peaks and the average content of the seven marker compounds (*P*_7*C*_) (see Table 5) for each sample. This good relationship demonstrates that the selected marker compounds (UR, AD, CGA, CFA, CCA, LGR and LG) basically synchronously changed with the overall content of the ISHI preparation chemicals. Therefore, quantitative evaluation of the fingerprints by SQFM has the potential to replace the use of multiple marker compounds and provides a reliable and feasible means to control the holistic quality consistency of ISHI.

### Correlation analysis between MEEKC fingerprint and antioxidant activities *in vitro*

#### Methodology validation of antioxidant activities

The absorbance of a single sample solution was continuously measured six times at 517 nm to assess the instrumental precision. The stability was tested by analyzing the DPPH solution stored at room temperature for 3 h. Six independent samples from the same batch were prepared and analyzed for the repeatability experiment. RSD(%) of the precision, stability and repeatability was 0.1%, 1.2% and 0.3%, respectively, indicating DPPH solution should be prepared as fresh as possible, and the method of antioxidant activities assay was reasonable and acceptable in 3 h.

#### Relationship between MEEKC fingerprint and antioxidant activities

The preliminary results of antioxidant activity (IC_50_) using DPPH method as described in Section “Antioxidant activity assay” were summarized in Table 5, and all of the measured rates (R^2)^ were above 0.99 (listed in Table A in S1 File). To explore the relationship between the MEEKC fingerprint and the antioxidant activities *in vitro* (Table 5), OPLS [34], a well-known chemometrics method, was performed by taking into the consideration relative peak area of the 27 co-possessing fingerprints as the descriptor matrix *X* and the 1/IC_50_ values as the response matrix *Y*. The 28 samples were divided randomly into two groups (Table 7) of the calibration and validation sets by bootstrap Latin partition method. Bootstrap Latin partition method divided 28 samples randomly into four equal parts (7 samples in each part), where three randomly parts (totally 21 samples) as calibration set and the other part (7 samples) as validation set. Replicates from the same sample will not be contained in the prediction and training sets at the same time, i.e. the validation samples were not included in the cross-validation. Five calibration-validation sets of the bootstrap Latin partition method were presented in Table B in S1 File, and “group 2” were adopted for the further OPLS study.

**Table 7:** Overview of the experimental and predicted values for total antioxidant activity of OPLS model.

Obs ID (Primary)	Y_Pred_ (1/IC_50_)	Y_Var_ (1/IC_50_)	RE (%) ^c)^
S1^a)^	0.350	0.351	0.40
S3 ^a)^	0.312	0.312	-0.01
S4 ^a)^	0.362	0.363	0.41
S5 ^a)^	0.355	0.354	-0.25
S6 ^a)^	0.314	0.315	0.16
S8 ^a)^	0.328	0.327	-0.39
S9 ^a)^	0.315	0.316	0.31
S10 ^a)^	0.331	0.329	-0.60
S12 ^a)^	0.330	0.331	0.31
S13 ^a)^	0.369	0.369	0.04
S14 ^a)^	0.352	0.353	0.22
S16 ^a)^	0.324	0.322	-0.64
S17 ^a)^	0.318	0.318	0.02
S19 ^a)^	0.368	0.369	0.18
S20 ^a)^	0.309	0.308	-0.48
S21 ^a)^	0.293	0.292	-0.18
S22 ^a)^	0.404	0.404	-0.09
S24 ^a)^	0.378	0.378	-0.07
S25 ^a)^	0.301	0.304	0.97
S27 ^a)^	0.423	0.423	-0.04
S28 ^a)^	0.328	0.327	-0.26
S2^b)^	0.311	0.312	0.43
S7 ^b)^	0.373	0.376	0.92
S11 ^b)^	0.330	0.335	1.37
S15 ^b)^	0.299	0.296	-0.94
S18 ^b)^	0.328	0.324	-1.17
S23 ^b)^	0.287	0.286	-0.48
S26^b)^	0.372	0.373	0.29

^a)^ Used for the calibration model.

^b)^ Used for the validation model.

^c)^ RE: relative error.

**Fig 5:**
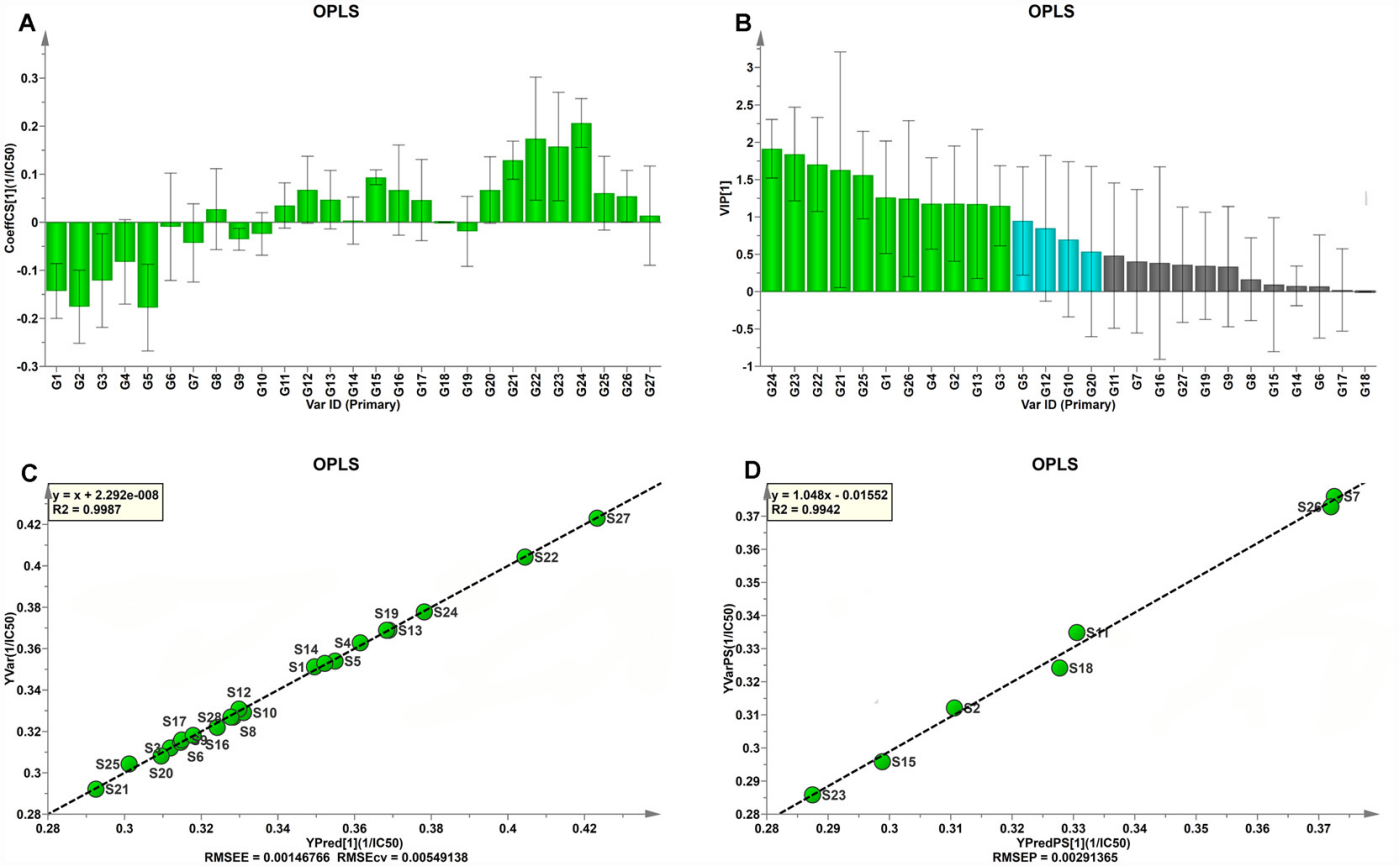
The OPLS model for ISHIs. (A) Coefficients plot. The bars indicate 95% confidence intervals based on jack-knifing; (B) VIP plot; (C) *Y* observed versus *Y* predicted plot for the calibration model; (D) *Y* observed versus *Y* predicted plot for the prediction model. Plots given by the SIMCA-P+ software (Version 13.0).

Form the obtained regression coefficients plot in Fig 5A, the majority of the MEEKC fingerprint components (including five identified peaks 12-LG, 13-LGR, 16-CGA, 22-CFA and 24-CCA) appears to have a positive influence and the rest peaks (including two identified peaks 6-AD and 10-UR) exhibiting negative correlation on the total antioxidant activity; indicating the antioxidants in ISHI may be phenolic acids and flavonoid compounds (CFA, CGA, CCA, LGR and LG) but not nucleoside (AD and UR). VIP values reflect the importance of variables in the model, the larger VIP, at least greater than 0.5, the more relevant for sample classification. It is obvious that the five identified peaks, i.e. 10, 12, 13, 22 and 24 with good confidence intervals, were the most relevant variables (Fig 5B).

The established calibration model achieved an explained variance (R^2^) of 99.87%, a predictive ability (Q^2^) of 97.30%, a root mean square error of estimation and a cross-validation procedure value of 0.0015 and 0.0055, respectively, indicating that the obtained model was optimal (Fig 5C). The established validation model was also applied to predict the antioxidant activity of samples in the validation sets. As showed in Fig 5D, an satisfactory result with an explained variance (R^2^) of 99.42% and a root mean square error of prediction value of 0.0029 were obtained, indicating that the established OPLS model possessed well predictive ability. From Table 7, no significant difference was observed between measured and predicted 1/IC_50_ values for all samples in calibration and prediction sets.

## Conclusions

In the present study, a high resolution MEEKC fingerprint of ISHI was successfully established. CCD response surface methodology was applied for the optimization of the MEs in MEEKC. The holistic quality consistency of the 28 batches of ISHI samples from two manufacturers was evaluated using the MEEKC fingerprint combined with the qualitative PCA and the seven-component quantitative determination. Furthermore, the qualities of the ISHI samples were well differentiated according to the SQFM from both the qualitative and quantitative aspects. In addition, the fingerprint—efficacy relationship between the fingerprint and the antioxidant activities was developed using the OPLS model, and the established model was an excellent predictor of antioxidant activity. The strategy proposed in this study is a reliable, effective and practical method that can be applied for the holistic quality assessment of TCM and herbal preparations.

## Supporting Information

S1 File Method A. Method of CZE. Method B. Method of MEKC. Figure A. Response surface plots for the interaction effects of the investigated variables and the measured *I* value. (A) *X*1*X*2 interaction at *X*3 = 7.5 mL, *X*4 = 4.0 mL, *X*5 = 3.0 mL (B) *X*1*X*3 interaction at *X*2 = 1.1 mL, *X*4 = 4.0 mL, *X*5 = 3.0 mL (C) *X*1*X*4 interaction at *X*2 = 1.1 mL, *X*3 = 7.5 mL, *X*5 = 3.0 mL (D) *X*1*X*5 interaction at *X*2 = 1.1 mL, *X*3 = 7.5 mL, *X*4 = 4.0 mL (E) *X*2*X*3 interaction at *X*1 = 305 mM, *X*4 = 4.0 mL, *X*5 = 3.0 mL (F) *X*2*X*4 interaction at *X*1 = 305 mM, *X*3 = 7.5 mL, *X*5 = 3.0 mL (G) *X*3*X*4 interaction at *X*1 = 305 mM, *X*2 = 1.1 mL, *X*5 = 3.0 mL (H) *X*3*X*5 interaction at *X*1 = 305 mM, *X*2 = 1.1 mL, *X*4 = 4.0 mL (I) *X*4*X*5 interaction at *X*1 = 305 mM, *X*2 = 1.1 mL, *X*3 = 7.5 mL. **Table A**. The calibration curve, R^2^ and IC_50_ (mg•mL^-1^) values of DPPH radical scavenging assay in 28 ISHI samples. **Table B**. Five calibration-validation sets of bootstrap Latin partition method.
(DOCX)
