# Supplementary material for: Microemulsion Electrokinetic Chromatography in Combination with Chemometric Methods to Evaluate the Holistic Quality Consistency and Predict the Antioxidant Activity of Ixeris sonchifolia (Bunge) Hance Injection
Source: PLoS One. 2016 Jun 23;11(6):e0157601. doi: 10.1371/journal.pone.0157601 (PMC4918936; doi:10.1371/journal.pone.0157601)
Supplement: S1 File — (A) X1X2 interaction at X3 = 7.5 mL, X4 = 4.0 mL, X5 = 3.0 mL (B) X1X3 interaction at X2 = 1.1 mL, X4 = 4.0 mL, X5 = 3.0 mL (C) X1X4 interaction at X2 = 1.1 mL, X3 = 7.5 mL, X5 = 3.0 mL (D) X1X5 interaction at X2 = 1.1 mL, X3 = 7.5 mL, X4 = 4.0 mL (E) X2X3 interaction at X1 = 305 mM, X4 = 4.0 mL, X5 = 3.0 mL (F) X2X4 interaction at X1 = 305 mM, X3 = 7.5 mL, X5 = 3.0 mL (G) X3X4 interaction at X1 = 305 mM, X2 = 1.1 mL, X5 = 3.0 mL (H) X3X5 interaction at X1 = 305 mM, X2 = 1.1 mL, X4 = 4.0 mL (I) X4X5 interaction at X1 = 305 mM, X2 = 1.1 mL, X3 = 7.5 mL. Table A. The calibration curve, R2 and IC50 (mg•mL-1) values of DPPH radical scavenging assay in 28 ISHI samples. Table B. Five calibration-validation sets of bootstrap Latin partition method. (DOCX) [file pone.0157601.s001.docx]

**Method A. Method of CZE**

The background electrophoretic of CZE was composed of 50 mM sodium tetraborate. The sample solutions were introduced into the capillary by hydrodynamic injection (10 cm height) for 20 s. The separation of ISHI was conducted at 16 kV at 25 ^o^C, and the detection wavelength was set at 210 nm.

**Method B. Method of MEKC**

The background electrophoretic of MEKC was composed of 50 mM sodium tetraborate and 20 mM SDS. The sample solutions were introduced into the capillary by hydrodynamic injection (10 cm height) for 20 s. The separation of ISHI was conducted at 16 kV at 25 ^o^C, and the detection wavelength was set at 210 nm.

**
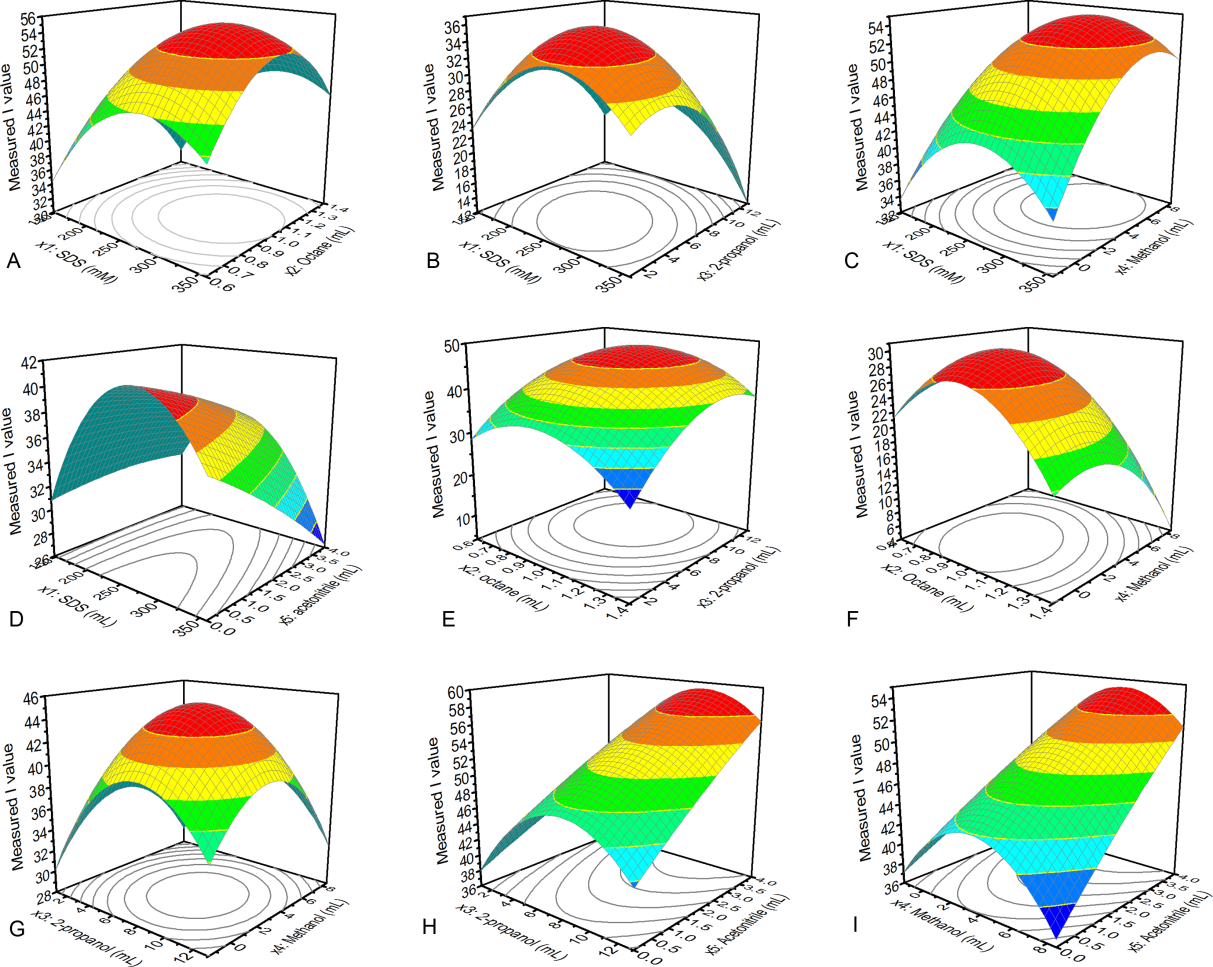
**

**Figure A.** **Response surface plots for the interaction effects of the investigated variables and the measured *I* value.** (A) *X*1*X*2 interaction at *X*3=7.5 mL, *X*4=4.0 mL, *X*5=3.0 mL (B) *X*1*X*3 interaction at *X*2=1.1 mL, *X*4=4.0 mL, *X*5=3.0 mL (C) *X*1*X*4 interaction at *X*2=1.1 mL, *X*3=7.5 mL, *X*5=3.0 mL (D) *X*1*X*5 interaction at *X*2=1.1 mL, *X*3=7.5 mL, *X*4=4.0 mL (E) *X*2*X*3 interaction at *X*1=305 mM, *X*4=4.0 mL, *X*5=3.0 mL (F) *X*2*X*4 interaction at *X*1=305 mM, *X*3=7.5 mL, *X*5=3.0 mL (G) *X*3*X*4 interaction at *X*1=305 mM, *X*2=1.1 mL, *X*5=3.0 mL (H) *X*3*X*5 interaction at *X*1=305 mM, *X*2=1.1 mL, *X*4=4.0 mL (I) *X*4*X*5 interaction at *X*1=305 mM, *X*2=1.1 mL, *X*3=7.5 mL.

**Table A**. The calibration curve, R^2^ and IC_50_ (mg·mL^-1^) values of DPPH radical scavenging assay in 28 ISHI samples.

| Sample | Calibration Curve | R^2^ | IC_50_ (mg·mL^-1^) |
| --- | --- | --- | --- |
| S1 | *y* = 18.6994*x* - 3.2934 | 0.9967 | 2.85 |
| S2 | *y* = 16.0427*x* - 1.4970 | 0.9961 | 3.21 |
| S3 | *y* = 14.1223*x* + 4.6673 | 0.9956 | 3.21 |
| S4 | *y* = 18.4587*x* - 0.7615 | 0.9946 | 2.75 |
| S5 | *y* = 18.4919*x* - 2.1471 | 0.9911 | 2.82 |
| S6 | *y* = 17.9136*x* - 6.7860 | 0.9973 | 3.17 |
| S7 | *y* = 19.4539*x* - 1.7474 | 0.9959 | 2.66 |
| S8 | *y* = 14.9146*x* + 4.3611 | 0.9912 | 3.06 |
| S9 | *y* = 15.9672*x* - 0.4656 | 0.9994 | 3.16 |
| S10 | *y* = 18.1031*x* - 5.0335 | 0.9929 | 3.04 |
| S11 | *y* = 15.2899*x* + 4.2831 | 0.9986 | 2.99 |
| S12 | *y* = 17.0152*x* - 1.386 | 0.9941 | 3.02 |
| S13 | *y* = 18.3093*x* + 0.3817 | 0.9977 | 2.71 |
| S14 | *y* = 16.0480*x* + 4.5841 | 0.9987 | 2.83 |
| S15 | *y* = 15.7663*x* - 3.2900 | 0.9938 | 3.38 |
| S16 | *y* = 13.5797*x* + 7.7672 | 0.9912 | 3.11 |
| S17 | *y* = 17.3417*x* - 4.4531 | 0.9945 | 3.14 |
| S18 | *y* = 18.4895*x* - 7.1325 | 0.9943 | 3.09 |
| S19 | *y* = 20.0231*x* - 4.2626 | 0.9914 | 2.71 |
| S20 | *y* = 16.1462*x* - 2.4753 | 0.9982 | 3.25 |
| S21 | *y* = 13.6178*x* + 3.4271 | 0.9961 | 3.42 |
| S22 | *y* = 20.8286*x* - 1.655 | 0.9978 | 2.48 |
| S23 | *y* = 14.0104*x* + 0.9635 | 0.9956 | 3.5 |
| S24 | *y* = 19.5565*x*-1.8248 | 0.9963 | 2.65 |
| S25 | *y* = 13.9401*x*+4.1371 | 0.9941 | 3.29 |
| S26 | *y* =16.4518*x* + 5.9091 | 0.9946 | 2.68 |
| S27 | *y* = 19.2383*x* + 4.5977 | 0.9923 | 2.36 |
| S28 | *y* = 18.4544*x* - 6.4706 | 0.9931 | 3.06 |

**Table B.** Five calibration-validation sets of bootstrap Latin partition method.

|  |  | 1 | 2 | 3 | 4 | 5 |
| --- | --- | --- | --- | --- | --- | --- |
| Calibration Set | | 2 ^f)^ 9 13 15 16 26 28 | 5 6 10 16 21 24 28 | 1 9 11 15 21 22 26 | 4 6 11 15 19 25 27 | 3 5 9 14 19 25 26 |
|  |  | 1 7 8 12 17 18 21 | 4 9 13 14 20 22 27 | 2 7 14 17 20 24 28 | 2 3 10 12 17 21 22 | 1 6 7 12 16 21 23 |
|  |  | 4 10 11 19 20 23 25 | 1 3 8 12 17 19 25 | 6 8 12 16 19 23 27 | 1 8 9 14 16 24 26 | 2 4 10 15 17 22 28 |
| Validation Set | | 3 5 6 14 22 24 27 | 2 7 11 15 18 23 26 | 3 4 5 10 13 18 25 | 5 7 13 18 20 23 28 | 8 11 13 18 20 24 27 |
| Calibration Model | R^2 a)^ | 99.87% | 99.87% | 99.94% | 99.91% | 99.44% |
|  | Q^2 b)^ | 97.20% | 97.30% | 98.00% | 97.60% | 95.70% |
|  | RMSEE ^c)^ | 0.0012 | 0.0015 | 0.0011 | 0.0013 | 0.0026 |
|  | RMSEcv ^d)^ | 0.0045 | 0.0055 | 0.0051 | 0.0053 | 0.0063 |
| Validation Model | R^2^ | 87.57% | 99.42% | 0.953 | 0.9706 | 0.9806 |
|  | RMSEP ^e)^ | 0.0129 | 0.0029 | 0.0064 | 0.0061 | 0.0055 |

^a)^ explained variance

^b)^ predictive ability

^c)^ root mean square error of estimation

^d)^ root mean square error of cross-validation

^e)^ root mean square error of prediction

^f)^ 1-28 represent sample S1-S28
